# Phenotypic and genomic comparisons of highly vancomycin-resistant *Staphylococcus aureus* strains developed from multiple clinical MRSA strains by *in vitro* mutagenesis

**DOI:** 10.1038/srep17092

**Published:** 2015-11-25

**Authors:** Kenichi Ishii, Fumiaki Tabuchi, Miki Matsuo, Keita Tatsuno, Tomoaki Sato, Mitsuhiro Okazaki, Hiroshi Hamamoto, Yasuhiko Matsumoto, Chikara Kaito, Tetsuji Aoyagi, Keiichi Hiramatsu, Mitsuo Kaku, Kyoji Moriya, Kazuhisa Sekimizu

**Affiliations:** 1 Laboratory of Microbiology, Graduate School of Pharmaceutical Sciences, The University of Tokyo, Tokyo, Japan.; 2 Department of Bacteriology, Faculty of Medicine, Juntendo University, Tokyo, Japan.; 3 Department of Infection Control and Prevention, Faculty of Medicine, The University of Tokyo, Tokyo, Japan.; 4 Department of Medical Technology, School of Health Sciences, Tokyo University of Technology, Tokyo, Japan.; 5 Department of Infection Control and Laboratory Diagnostics, Internal Medicine, Tohoku University Graduate School of Medicine, Tokyo, Japan.

## Abstract

The development of vancomycin (VCM) resistance in *Staphylococcus aureus* threatens global health. Studies of the VCM-resistance mechanism and alternative therapeutic strategies are urgently needed. We mutagenized *S. aureus* laboratory strains and methicillin-resistant *S. aureus* (MRSA) with ethyl methanesulfonate, and isolated mutants that exhibited high resistance to VCM (minimum inhibitory concentration = 32 μg/ml). These VCM-resistant strains were sensitive to linezolid and rifampicin, and partly to arbekacin and daptomycin. Beta-lactams had synergistic effects with VCM against these mutants. VCM-resistant strains exhibited a 2-fold increase in the cell wall thickness. Several genes were commonly mutated among the highly VCM-resistant mutants. These findings suggest that MRSA has a potential to develop high VCM resistance with cell wall thickening by the accumulation of mutations.

The discovery of antibiotics has equipped humans with potent weapons to combat infectious pathogens, but antibiotic-resistant mutants continue to emerge. Methicillin-resistant *Staphylococcus aureus* (MRSA) is one of the most notorious life-threatening drug-resistant pathogens^1^. Vancomycin (VCM) is used to treat patients with MRSA in hospitals throughout the world. Mutants resistant to VCM have recently emerged, however, due to its misuse; strains are classified by the resistance level as VCM-intermediate resistant *S. aureus* (VISA; MIC = 4–8 μg/ml) or VCM-resistant *S. aureus* (VRSA; MIC ≥16 μg/ml) according to the Clinical and Laboratory Standards Institute^2^. Although there was an early report of VRSA with high VCM MIC developed in a laboratory^3^, the phenotypic and genetic characteristics of the highly VCM-resistant strains were not fully elucidated.

In the early 2000s, researchers reported that *S. aureus* strains acquiring the *vanA* gene by transposon and plasmid transfer from VCM-resistant *Enterococcus* showed an extremely high MIC to VCM^4^,^5^. This *vanA*-type of VRSA has been isolated in relatively restricted areas and has not yet become an overwhelming majority^6^. Instead, increasing reports indicate the globally widespread occurrence of VISA, which has likely developed VCM resistance by accumulating mutations rather than exogenously gaining the resistance cassette^7^. Genome-wide analysis and genetic manipulations using clinical isolates have begun to reveal the mechanisms underlying the progression of VCM-resistance in VISA, which involve mutations in several genes, including *rpoB* (encoding a subunit of RNA polymerase), *pp2c* (encoding a protein phosphatase), *graSR*, *walKR*, and *vraSR* (encoding two-component regulatory systems)^8^,^9^,^10^,^11^,^12^,^13^,^14^,^15^,^16^. On the other hand, VRSA strains showing VCM MIC values higher than 16 μg/ml are rarely isolated from clinical specimens, and therefore the mechanism underlying progression of the development of high VCM resistance remains to be clarified.

In this report, we mutagenized *S. aureus* laboratory strains and clinical isolates to facilitate the development of resistance and selected highly VCM-resistant cells. Moreover, we characterized the phenotypes, including the antibiotic susceptibility profiles, cell structure morphologies, and gene mutations of these VCM-resistant strains.

## Results

### Selection of VCM-resistant mutants from *S. aureus* clinical strains

**Figure 1:**
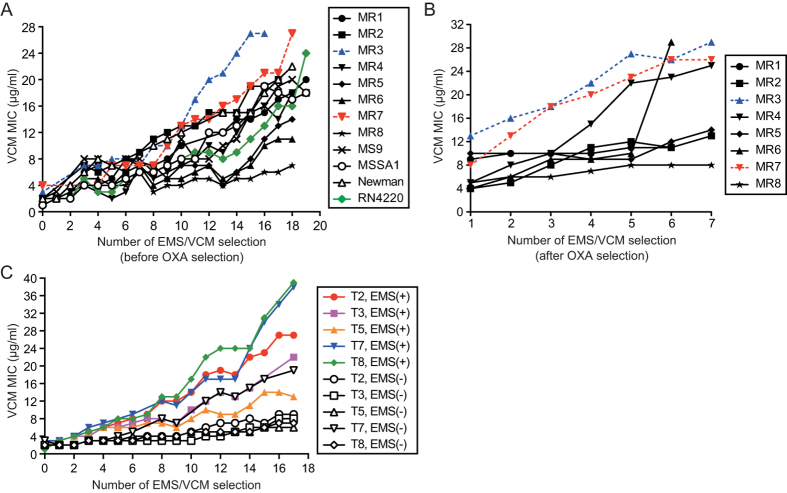
Increase in VCM-resistance of *S. aureus* through EMS mutagenesis. (**A**) *S. aureus* strains including clinical isolates (MR1-MR8 and MS9 isolated at the University of Tokyo hospital) and laboratory strains were cultured in the presence of 0.1% EMS. Each overnight culture was inoculated in EMS-free broth for an outgrowth, and VCM-resistant mutants were selected. MIC values were determined as described in the Methods. (**B**) Among the VCM-resistant mutants obtained in A, those that originated from MRSA were passaged with 32 to 128 μg/ml of OXA. The selected OXA-resistant mutants were continuously subjected to EMS/VCM selection. Number of EMS/VCM selections after the OXA passage is shown in the x-axis. (**C**) Five MRSA strains clinically isolated at Tohoku University hospital^36^ were cultured with (closed symbols) or without (open symbols) 0.1% EMS. VCM-resistant mutants were selected as described in A.

We first attempted to obtain VCM-resistant *S. aureus* strains by serial mutagenesis using RN4220, a methicillin-sensitive laboratory strain. The MIC values against VCM gradually increased with successive ethyl methanesulfonate (EMS) treatment and selection in VCM-containing broth (Fig. 1). We then applied this mutagenic approach to clinical MRSA independently isolated from different regional hospitals in Japan. Regardless of the origin, the VCM MIC of all clinical strains increased due to the EMS/VCM selection (Fig. 1). After more than 10 rounds of VCM selection, most of the MRSA-derived mutants exhibited increased oxacillin (OXA) sensitivity (OXA MIC ≤4 μg/ml), except those derived from MR1 (OXA MIC = 128 μg/ml), MR4 (OXA MIC = 8 μg/ml), and MR5 (OXA MIC >128 μg/ml). This finding indicates that VCM selection tended to abolish the original property of the five MRSA strains (*i.e.*, resistance to beta-lactams), which was previously reported as a “seesaw effect”^17^. In the course of characterizing the phenotypes of the VCM-resistant strains in OXA-containing Mueller-Hinton broth, we observed an OXA-resistant bacterial population within the OXA-sensitive population. Therefore, we isolated the OXA-resistant cells by passage with 32 to 128 μg/ml of OXA, and further isolated VCM-resistant strains. After a total of 20 to 25 rounds of EMS/VCM selection, we obtained MRSA-derived mutants with high MIC values to both VCM and OXA, based on the micro-dilution method in liquid medium (Table 1). In addition, we confirmed the high VCM- and OXA-resistance of these mutants by the E-test (*i.e.*, a method to determine MIC values on agar plates with strips containing a gradient concentration of each antibiotic^18^) (Supplemental Table S2). Colonies isolated by EMS/VCM selection from methicillin-sensitive strains (*i.e.*, MS9, MSSA1, Newman, and RN4220) also exhibited high VCM MIC values (Fig. 1A, Table 1). This finding supported previous notions that SCC*mec* regions of MRSA are dispensable for acquiring the VCM-resistant phenotype^19^.

Mutants named VR3 and VR7, originating from clinical isolates MR3 and MR7, respectively, had MIC values of 32 μg/ml VCM and ≥128 μg/ml OXA (Table 1). The VCM-resistant mutants, including VR3 and VR7, had longer doubling times than the parent strains (Supplemental Table S3). In the course of MIC analyses using the E-test, we observed heterogeneous-sized colonies in some strains, including VR3, VR6, and VR7. The appearance rate of the large colonies in VR3 and VR7 under drug-free conditions was 9% and 14%, whereas that in VR-RN was less than 0.1%. Isolates from the large colonies (L1 and L2 strains shown in Supplemental Table S3) had lower VCM MIC values than each parent strain.

**Figure 2:**
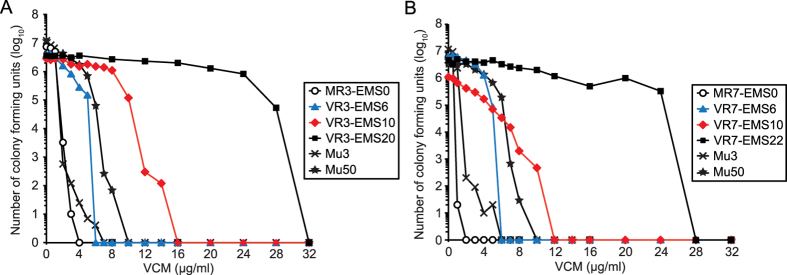
Population analysis of the VCM-resistant strains. The parent MRSA clinical isolates (MR3-EMS0 (**A**) or MR7-EMS0 (**B**)), the intermediate VCM-resistant strains isolated after 6 or 10 rounds of EMS/VCM selection (VR3-EMS6, VR3-EMS10, VR7-EMS6, or VR7-EMS10), the VCM-resistant mutants isolated after 20 or 22 rounds of EMS/VCM selection (VR3-EMS20 or VR7-EMS22), and VISA strains (Mu3 and Mu50) were grown in BHI broth. Each overnight culture was diluted to OD_576_ of 0.3, and 10-fold serial dilution was spread on BHI agar plates containing the indicated VCM concentrations. Colonies were counted after incubation at 37 °C for 72 h.

Previous studies demonstrated that Mu3 and Mu50, clinically isolated as heterogeneous VISA (hVISA) and VISA, respectively, contained populations with heterologous resistance to VCM^20^. To examine the heterogeneity within the above VCM-resistant mutants, we used intermediate VCM-resistant mutants with MICs of 8 or 16 μg/ml isolated during the interim EMS/VCM selection. In this section, the original MRSA strains are indicated as “MR3-EMS0” and “MR7-EMS0”, and the VCM-resistant mutants isolated after 20 or 22 rounds of EMS/VCM selection (characterized in the above section as VR3 and VR7) are indicated as “VR3-EMS20” or “VR7-EMS22”. Strains named “VR3-EMS6”, “VR3-EMS10”, “VR7-EMS6”, and “VR7-EMS10” represent intermediate mutants isolated after 6 or 10 rounds of EMS/VCM selection originating from either MR3 or MR7, respectively. Overnight cultures were diluted and spread on agar plates containing various concentrations of VCM. After incubation for 72 h, we counted the numbers of colonies on the plates. The mutants VR3-EMS20 and VR7-EMS22 formed colonies on agar plates with 28 and 24 μg/ml VCM, respectively (Fig. 2). Consistent with their MIC values, VR3-EMS6, VR3-EMS10, VR7-EMS6, and VR7-EMS10 mutants showed intermediate phenotypes of VCM resistance (Fig. 2, Supplemental Table S3). Moreover, heterologous VCM-resistant populations were observed in VR3-EMS10 and VR7-EMS10 mutants as well as in Mu3 and Mu50 (Fig. 2). Furthermore, we examined the VCM-resistant phenotype of cells that formed colonies on the VCM-containing agar plates during the population analysis. We isolated 3 colonies each from 8, 16, or 28 μg/ml VCM plates spread with VR3-EMS20 and 3 colonies each from 8, 16, or 24 μg/ml VCM plates spread with VR7-EMS22. The VCM MIC values, determined by the micro-dilution method after 48 h incubation, of the isolated colonies were all 32 μg/ml, confirming that the cells themselves were resistant to VCM (and were not VCM-sensitive satellite colonies). Therefore, we concluded that *S. aureus* clinical isolates could develop high VCM-resistance through successive EMS/VCM selection.

### Susceptibilities of the VCM-resistant strains to other antibiotics

We next examined the susceptibilities of the VCM-resistant strains to other antibiotics clinically used to treat MRSA infection, such as arbekacin, linezolid, daptomycin, gentamicin, and rifampicin. We also tested the effects of lysocin E, a novel antibiotic that we recently identified, whose target was determined to be menaquinone within the bacterial membrane^21^. We found that the VCM-resistant mutants had MIC values comparable to those of the parent strains, except for the elevated MIC values of arbekacin and daptomycin against VR7 (Table 2). These findings suggest that the increased level of VCM resistance acquired through repeated EMS-treatment and VCM-selection is not associated with a multidrug-resistant phenotype.

**Figure 3:**
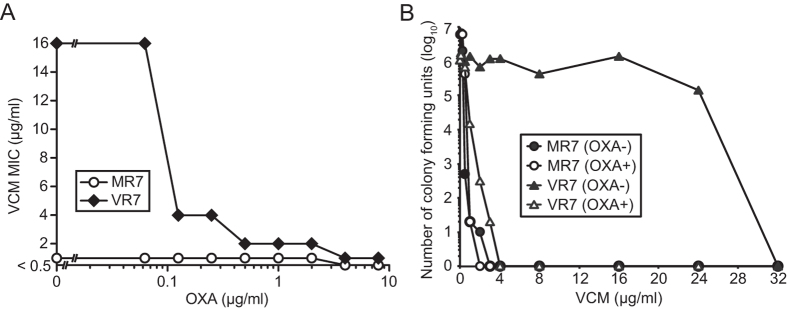
Synergistic effect of beta-lactam antibiotic and VCM against the VCM-resistant strain. (**A**) Dose response of OXA for the synergistic effect with VCM. MIC values of VCM in the presence of various concentrations of OXA were determined by the micro-dilution method after 48 h incubation. (**B**) Population analysis in the presence of OXA. Cultures of either the parent MRSA clinical isolate MR7 or the VCM-resistant mutant VR7 were spread on BHI agar plates containing VCM at the indicated concentrations with or without OXA (2 μg/ml). Colonies were counted after incubation at 37 °C for 72 h.

Previous reports suggest that beta-lactam antibiotics act cooperatively with VCM against some clinically-isolated VISA strains^22^. We next tested the effects of beta-lactams on the action of VCM against the VCM-resistant strains. Addition of OXA at 2 μg/ml, a concentration much lower than the MIC (>128 μg/ml), led to a striking increase in the VCM sensitivity of VR3 and VR7. The VCM MICs of VR3 and VR7 in the presence of 2 μg/ml OXA were indistinguishable from those of the parent strains (Table 3). Cefazolin, another beta-lactam antibiotic, also increased the VCM sensitivity against these VCM-resistant mutants, whereas an aminoglycoside, gentamicin, did not, suggesting that the synergy with VCM against these VCM-resistant strains was specific for beta-lactams (Table 3). The VCM sensitivity of VR7 was increased by addition of OXA (≥ 0.125 μg/ml) in a dose-dependent manner (Fig. 3A). Moreover, population analysis of VR7 in the presence of 2 μg/ml OXA demonstrated that the presence of OXA dramatically increased the sensitivity of this mutant to VCM (Fig. 3B). These findings suggest that beta-lactam antibiotics synergistically act with VCM on the VCM-resistant mutants isolated in this study.

### Cell wall structures of the VCM-resistant strains

**Figure 4:**
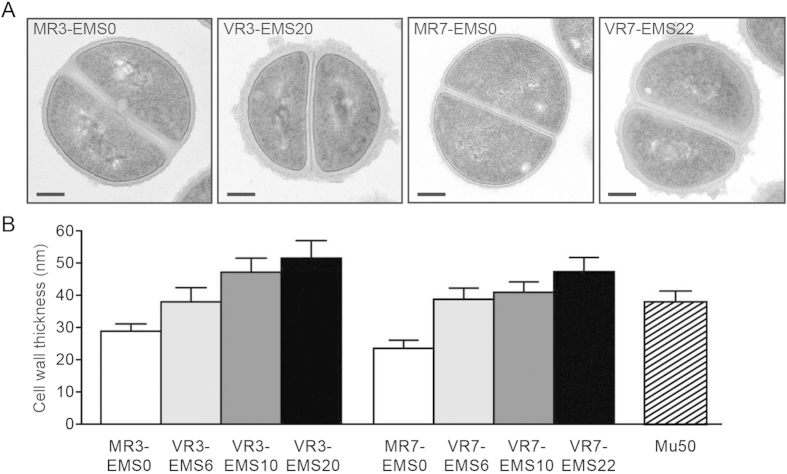
Transmission electron microscopy photographs of VCM-resistant strains. (**A**) Representative photograph of each strain. Scale bar: 200 nm. (**B**) Cell wall thickness of each strain. Data represent mean ± SD of 30–41 cells. Statistical significance was analyzed within each lineage by one-way ANOVA with Tukey’s multi-comparison test. P values between the MR3-originated strains are all under 0.001. P values between the MR7-originated strains are under 0.001, except for that between VR7-EMS6 and VR7-EMS10, which is under 0.05.

Previous reports demonstrated that thickened cell walls are a common feature of VISA^17^,^23^. We used transmission electron microscopy to analyze the cell wall structures of the VCM-resistant mutants developed from MR3 and MR7. The parent MR3-EMS0 and MR7-EMS0 strains had smooth surfaces (Fig. 4A) with cell wall thicknesses of 28.9 ± 2.2 nm and 23.5 ± 2.5 nm, respectively (Fig. 4B). In contrast, the VCM-resistant strains exhibited rough and fluffy surfaces (Fig. 4A), and the cell wall thickness increased with the number of EMS/VCM selection rounds (Fig. 4B). Mean cell wall thicknesses of VR3-EMS20 and VR7-EMS22 (51.5 ± 5.4 nm and 46.6 ± 3.9 nm, respectively) increased by 1.8- and 2.0-fold compared with the respective parent strain. These findings suggest that the accumulation of mutations leads to cell wall thickening associated with VCM resistance.

### Identification of mutated genes in the VCM-resistant strains

As for genetic characterization of the isolated VCM-resistant strains, we performed whole genome sequencing analysis using next generation sequencing. We analyzed the genomes of seven sets of MRSA-originating strains (MR/VR1, 2, 3, 4, 5, 7, 8) and a set of RN4220-originating strains (RN4220 and VR-RN). Multi-locus sequence typing of each MRSA strain allowed us to categorize five strains (MR1, 3, 5, 7, 8) as ST5, and two strains (MR2, 4) as ST8. Because N315 and USA300 are representative strains of ST5 and ST8, respectively, for which the genomes have been characterized, we used them as reference genomes. Each genome sequence was compared with the reference genome, and the difference within coding regions between each set of strains was then extracted. Throughout the study, we focused on gene mutations that caused non-synonymous changes, and synonymous mutations were therefore omitted. We found that a number of genes ranging from 50 to 172 were mutated in the VCM-resistant strains compared with each parent strain. This finding indicates that an average of 2 to 7 mutations in the coding regions were induced by each step of EMS/VCM selection. Among them, one gene (SA0615; *graS*) was commonly mutated in five of eight strain sets (Table 4). Seven genes, including SA0500 (*rpoB*) and SA0501 (*rpoC*), previously reported to be involved in VCM-resistance^8^,^9^,^12^, were mutated in four of eight strain sets (Table 4). In addition, five genes, including SA0018 (*walK*), another gene reported in clinical VISA^11^,^14^, were commonly found in three strain sets (Table 4). Furthermore, we compared the mutation patterns within the high-MIC (VCM MIC = 32 μg/ml; VR3, 4, 7 and VR-RN) group. Among 323 genes mutated in the high-MIC group, 277 genes were mutated only in the high-MIC group and not in the other strains. Three genes (SA0173 [*ausA*], SA0519 [*sdrC*], and SA1584 [encoding lysophospholipase L2]) that have no previously reported relationship with VCM-resistance, were commonly mutated in 3 high-MIC strains, and 17 genes (including genes related to VCM-resistance such as *walK* and *graS*^10^,^11^,^24^,^25^) were commonly mutated in 2 high-MIC strains (Table 4).

We further analyzed the sequence data of the eight VCM-resistant strains to examine whether the mutations were associated with the functional categories. In general, half of the mutants (4 of 8 strains) had the highest mutation frequencies in the “amino acid transport and metabolism”-category genes (Supplemental Table S4). Comparison between low- (MIC = 8 μg/ml; VR1, 2, and 5) and high-MIC (MIC = 32 μg/ml; VR3, 4, 7, RN) groups did not reveal prominent differences (Supplemental Table S4). We found modest variations in the mutation frequencies for the “signal transduction mechanisms”, “energy production and conversion”, and “inorganic ion transport and metabolism” categories, which were slightly higher in the high-MIC group than in the low-MIC group (Supplemental Table S4).

**Figure 5:**
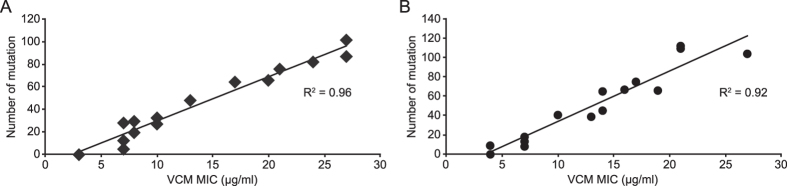
Correlation between the VCM MIC and number of mutations in the VCM-resistant strains. Whole genome sequencing was performed for the intermediate strains obtained during the EMS/VCM selection. As for MR3- (**A**) or MR7-derived strains (**B**), mutants isolated after 3–16 rounds (14 strains, from VR3-EMS3 to VR3-EMS16) or 3–18 rounds (16 strains, from VR7-EMS3 to VR7-EMS18) were analyzed. The VCM MIC values, determined as described in the Methods, and the number of mutations in each intermediate strain was plotted. The R^2^ value indicates the correlation coefficient between the VCM MIC and number of mutations.

We then focused on two high VCM-resistant strains and performed additional genomic analyses regarding the intermediate isolates developed from MR3 (14 strains, from VR3-EMS3 to VR3-EMS16; Supplemental Table S5) and MR7 (16 strains, from VR7-EMS3 to VR7-EMS18; Supplemental Table S6). Plotting the number of non-synonymous mutations and VCM MIC resulted in correlation coefficients (R^2^ values) of 0.96 in MR3- and 0.92 in MR7-originating strains, respectively (Fig. 5). These results suggest that the number of mutations is positively correlated with the increase in VCM MIC, and support the previous reports demonstrating a similar trend in VISA^26^,^27^.

## Discussion

The history of antibiotic development is associated with the emergence of dissidents that evade therapeutic treatment. Pathogens are apparently equipped with the potential to develop drug resistance as an evolutionary process for their adaptation to the external environment. Therefore, elucidation of antibiotic-resistant mechanisms will enhance the development of clinical remedies as well as our understanding of basic evolutionary aspects. In this report, we first developed *S. aureus* strains highly resistant to VCM, first from laboratory strains and then from clinical isolates, and then characterized their phenotypes. To the best of our knowledge, this is the first report of an experimental development of highly VCM-resistant *S. aureus* mutants from several different origins followed by their genomic comparisons.

Most artificially generated VCM-resistant strains lose their original MRSA properties and became susceptible to OXA and other beta-lactams, a phenomenon called the “seesaw effect”^3^,^17^, for which the precise mechanism is currently unknown. Under our experimental conditions, we also observed a decrease in the OXA MIC during EMS/VCM selection in cells originating from MRSA strains. As we assumed that strains resistant to both VCM and beta-lactams could be truly problematic clinically, we attempted to select strains with high resistance to both VCM and OXA for further characterization. While the MRSA-originating mutants were resistant to VCM and OXA, they were sensitive to other antibiotics with different antimicrobial mechanisms, such as linezolid and rifampicin. In addition, these VCM-resistant strains exhibited an unaltered susceptibility against lysocin E, a novel menaquinone-targeting antibiotic that we recently identified^21^. One of the VCM-resistant strains, VR7, exhibited increased resistance to daptomycin compared with the parent, MR7. This phenotype of VCM and daptomycin resistance is typically observed in VISA^14^,^28^. Regardless of the differences in antibiotic susceptibilities between strains, the combined use of OXA or cefazolin with VCM led to a potent antibacterial effect even against highly VCM-resistant mutants, as reported for VISA^22^. As suggested in a previous report, beta-lactams likely reduce cell wall thickness, leading to reduced binding of VCM with cell wall targets^22^. Despite the almost 2-fold increase in cell wall thicknesses of VR3 and VR7 compared with the respective parent strains, the combined use of VCM and beta-lactams exhibited efficient antibacterial effects. Because the addition of gentamicin failed to increase VCM susceptibility, the synergistic effect with VCM was specific for beta-lactams, which requires close attention with regard to clinical antibiotic selection. In preparation for the future outbreak of pathogens more highly resistant to VCM than the present prevalent VISA, the mutants isolated in this study will provide useful perspectives for alternative therapeutic approaches.

Recently, Berscheid and colleagues reported the experimental development of VCM-resistant strains^29^. They constructed a deletion mutant of the *mutS* gene, which encodes a DNA repair factor, in the RN4220-strain background. Using this ∆*mutS* mutant having an increased mutation rate, they obtained VCM-resistant mutants through successive passages and demonstrated that mutations in the *vraS* gene were partially involved in the acquisition of the VISA phenotype^29^. The genetic mutations involved in the processes to achieve high resistance to VCM, however, have not yet been determined. To gain insight into the developmental processes of high VCM resistance, we further selected VCM-resistant cells originating from several independent parents and compared the mutation profiles within the high-MIC group. Based on our findings that the mutated genes did not completely overlap within the high-MIC group, we considered that the mechanisms underlying the development of VCM resistance were not the same. Because critical steps that led to sharp increases in MIC values were hardly observed during the EMS/VCM selection (Fig. 1) and the accumulation of multiple mutations was positively correlated with VCM resistance (Fig. 5), the contribution of most single mutations to VCM resistance could be barely detectable. In addition, as the mutants exhibit significant physiological alterations (*i.e.*, growth defects and cell wall thickening), the mutations seem to be selected according to the complex balance of fitness cost. Comparison of the genome among the intermediate strains revealed that several mutations failed to be carried over to the next step of EMS/VCM selection (Supplemental Tables S5, S6), suggesting that mutations fixed over the series of selection constitute distinct combinations required for bacterial survival in the presence of high concentrations of VCM. As a result, the mutation combinations appeared to vary among the VCM-resistant strains isolated after 19–25 rounds of EMS/VCM selection (Table 4). We considered this variability to be a genetic characteristic of *S. aureus* mutants adapted to high concentrations of VCM.

Besides one mutation (H134Y) in the SA1678 (*perR*) gene commonly detected in two high VCM-resistant strains (VR3 and VR7), the rest of the mutation regions varied among the strains (Table 4). Although the mutated sites were not identical among mutants, several strains had the same genes mutated. These coincident mutations of the same genes are not likely to be caused by chance for the following reason: given that the mean number of mutation caused by a single EMS treatment is ten^30^ and that *S. aureus* possesses a total of 3 × 10^3^ genes, the possibility that of a gene being mutated is 1/300 (if all mutations are caused in coding regions without overlap). Under the same conditions, the possibility that of a gene being mutated after 20 rounds of EMS treatment is 1/15. Then, the possibilities that the same gene being mutated among 3, 4, and 5 independent strains are (1/15)^3^, (1/15)^4^, and (1/15)^5^, respectively. By random selection, the numbers of mutated genes commonly observed in 3, 4, and 5 strains would be less than 1 (calculated as 0.9, 0.06, and 0.004, respectively). Based on the above estimate and the consistency of our results with previous reports of VCM-resistance relationship in some genes, we consider that the genes we identified in the present study could be involved in acquiring the high VCM resistance in *S. aureus*. We therefore considered the possibility that various mutations could cause similar effects on each gene product (e.g., conformational changes and alterations of enzymatic activities) that contribute to the resistant phenotype.

Although not common to all four high-MIC strains, we identified three genes, *ausA*, *sdrC*, and SA1584, that were mutated in three of four strains. While these genes were in part reported as virulence factors, their involvement in VCM resistance has not been documented. The *ausA* gene product is involved in the production of a peptide secondary metabolite of *S. aureus* called aureusimine. The first study of aureusimine demonstrated its involvement in *S. aureus* virulence^31^, which was followed by an opposing report from another research group^32^. The second *sdrC* gene encodes an adhesive molecule at the bacterial surface and contributes to the virulence potential^33^. We found two of three mutations (S746L in VR3 strain and D821N in VR-RN strain) within the repetitive regions (from the 718th to 893th amino acids) and one positioned in the carboxypeptidase regulatory-like domain (from the 625th to 685th amino acids). Alterations of the cell surface proteins might affect the accessibility of VCM to the target site. The third gene, SA1584, encodes a putative lysophospholipase L2 involved in phospholipid turnover, as reported in *E. coli*^34^. The altered phospholipid composition in the cytoplasmic membrane of *S. aureus* is associated with resistance against antibiotics, including VCM^35^. In addition, we found several mutated genes that were previously reported in VISA, such as *graS*, *walK*, *rpoB*, and *rpoC*. The phenotypes of the highly VCM-resistant cells that we described in the present study share common features with those of the VISA strain harboring mutations in *walK*^11^ and *rpoB*^8^,^9^,^12^, such as cell wall thickening and growth defects. Because such genes known to be associated with VISA phenotypes are also mutated, we consider that our *in vitro* mutagenic approach to generate highly VCM-resistant strains reflects some aspects of future evolution in VCM resistance beyond VISA. One mutation site of the *rpoB* gene (the 406th arginine) in VR1 was the same as that previously described in VISA^9^,^27^, whereas the other sites were not. This suggests that the developmental paths to achieve high VCM resistance could vary, as discussed above. Although the actual mutagenic mechanisms and conditions are not identical to those of artificial EMS treatment, the host-invading bacteria are under continuous attack, not only by administered antibiotics but also by the immune system and various stresses (*e.g.*, oxidative stress) that are likely to promote mutation and selection. By focusing on the above genes, including the VISA-related genes and those that have not yet gained focus in the context of VCM resistance, future analyses of the relationship between each gene mutation and resistance are required to clarify the evolutionary pathways to the development of high resistance to VCM.

## Materials and Methods

### Bacterial strains

MRSA strain Nos. MR1-MR8 and MSSA strain No. MS9 were isolated from human blood samples at the University of Tokyo Hospital. Other *S. aureus* strains were previously characterized, and the strain information is summarized in Supplemental Table S1. In all experiments, bacteria were incubated at 37 °C.

### Mutagenesis and selection of antibiotic resistant strains

*S. aureus* laboratory strains and clinical isolates were cultured in tryptic soy broth (TSB). Two microliters of each overnight culture was inoculated to 1 ml of TSB containing 0.1% EMS, and aerobically incubated for 12 to 24 h. Fifty microliters of each suspension was transferred to 1 ml fresh (EMS-free) TSB, and incubated for another 12 to 24 h for outgrowth. The suspensions were diluted 1000-fold with TSB containing various concentrations of VCM, followed by static incubation for 2 days. Among the wells in which bacterial growth was not observed, the lowest VCM concentration was determined as the MIC. To avoid confusion with conventional methods, such as the micro-dilution method and E-test, MIC values determined in this manner are mentioned as such in the figure/table legends. Suspended bacteria obtained from the sub-MIC wells were subjected to the next round of EMS mutagenesis, and the VCM selection procedures were repeated. After 19 to 26 rounds of EMS treatment, each outgrowth culture was spread on agar plates containing VCM at concentrations around the MIC values. After 2 to 3 days incubation, colonies were isolated and named as VR1-9 and VR-RN strains.

### Antimicrobial assays

MIC assays were performed by the micro-dilution method as previously described^21^. Briefly, bacterial cells were scraped from agar plates and suspended in sterilized saline at OD_600_ = 0.5. The bacterial suspensions were diluted 400-fold with Mueller-Hinton broth and transferred to 96-well round bottom plates. Serial dilutions of antibiotics were added to each well. Plates were incubated at 37 °C, and MIC values were determined after 48 h.

An E-test was performed essentially as described in a previous report^18^ using strips of VCM and OXA (bioMerieux). Because of the slow growth phenotypes of the mutants, the MIC values were determined on Mueller-Hinton agar after 72 h of incubation at 37 °C, instead of 24 h, according to a previous report using slow-growing VISA strains^12^.

Population analysis was performed according to a previous report^12^. The colony forming units (CFUs) on brain heart infusion (BHI) agar containing various concentrations of VCM were determined after 72 h incubation.

### Transmission electron microscopy

Cells grown to mid-log phase (OD_576_ = 0.5–0.6) were harvested and fixed by 2% glutaraldehyde. Photographs were obtained under the transmission electron microscope and cell wall thickness was measured as described in a previous report^9^.

### Genome sequencing and comparison analysis

Genome DNA of each strain was extracted using a QIAamp DNA Blood Mini Kit (Qiagen). The 250-bp paired-end read sequencing of seven sets of MRSA-originating strains (MR/VR1, 2, 3, 4, 5, 7, 8) and a set of RN4220-originating strains (RN4220 and VR-RN) was performed using the MiSeq sequencing platform (Illumina) according to a previous report^12^. Comparison of genome sequences using CLC Genomics Workbench 7.5 (CLC bio) was performed as follows. First, each genome sequence was compared with the reference genome. The paired genome sequences of each MRSA strain was subjected to the multi-locus sequence typing analysis using a program provided by Center of Genomic Epidemiology (http://www.genomicepidemiology.org). Five strains (MR1, 3, 5, 7, 8) were categorized as ST5, and two strains (MR2, 4) were assigned as ST8. Because N315 (accession No. NC_002745.2) and USA300_FPR3757 (accession No. NC_007793.1) are representative strains of ST5 and ST8, respectively, whose genomes have been characterized, they were used for reference genomes. Second, the difference within coding regions between each set of strains was extracted.

## Additional Information

**How to cite this article**: Ishii, K. *et al.* Phenotypic and genomic comparisons of highly vancomycin-resistant *Staphylococcus aureus* strains developed from multiple clinical MRSA strains by *in vitro* mutagenesis. *Sci. Rep.* **5**, 17092; doi: 10.1038/srep17092 (2015).

## Supplementary Material

Supplementary Information

Supplementary Dataset 1

Supplementary Dataset 2

Supplementary Dataset 3

## Figures and Tables

**Table 1:** MIC values of VCM and OXA against the parent and mutant strains.

Strain	VCM	OXA	Strain	VCM	OXA
MR1	2	>128	VR1	8	>128
MR2	1	128	VR2	8	>128
MR3	2	>128	VR3	32	>128
MR4	1	16	VR4	32	128
MR5	2	>128	VR5	8	>128
MR6	1	64	VR6	16	>128
MR7	1	>128	VR7	32	128
MR8	1	>128	VR8	16	>28
MS9	2	<2	VR-MS9	32	<2
RN4220	<0.5	<2	VR-RN	32	<2

MIC values were determined after 48 h incubation at 37 °C by the micro-dilution method. VCM, vancomycin; OXA, oxacillin. Unit: μg/ml.

**Table 2:** MIC values of antibiotics against the VCM-resistant strains.

Strain	VCM	OXA	LYE	ABK	LNZ	DAP	GTM	RFP
MR3	2	>128	8	2	4	0.5	1	<0.25
VR3	32	>128	8	1	2	1	1	<0.25
MR7	1	>128	8	4	4	0.5	>16	<0.25
VR7	32	>128	8	16	2	8	>16	<0.25

MIC values were determined after 48 h incubation at 37 °C by the micro-dilution method. VCM, vancomycin; OXA, oxacillin; LYE, lysocin E; ABK, arbekacin; LNZ, linezolid; DAP, daptomycin; GTM, gentamicin; RFP, rifampicin. Unit: μg/ml.

**Table 3:** Synergistic effects of beta-lactams and VCM against the VCM-resistant strains.

Strain	VCM	VCM + OXA	VCM + CFZ	VCM + GTM
MR3	1	1	1	1
VR3	32	2	2	32
MR7	1	1	1	1
VR7	32	2	1	32

The concentrations of OXA and CFZ were fixed to 2 μg/ml, and the MIC values of VCM are shown. The concentrations of GTM used for MR3 and VR3 were 0.25 μg/ml, and those for MR7 and VR7 were 8 μg/ml. Unit: μg/ml.

**Table 4:**
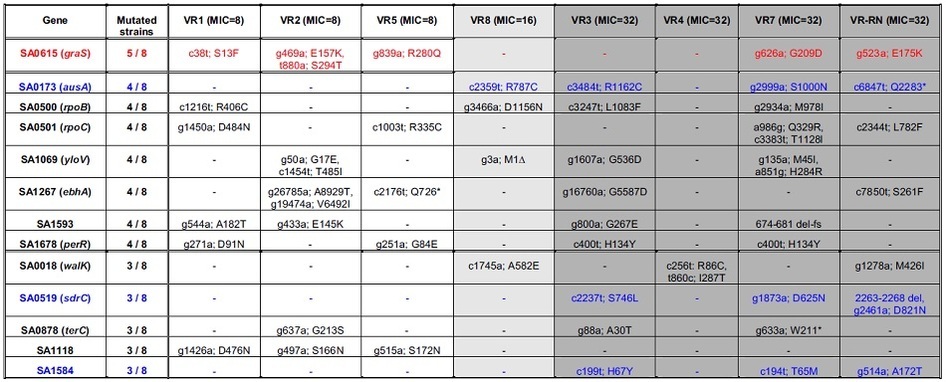
Mutated genes commonly observed among the VCM-resistant strains and the respective mutation site.

Mutated genes found commonly in three or more VCM-resistant strains are shown. The VCM MIC value of each mutant is shown in the top row within parentheses (unit: μg/ml). “Mutated strains” indicate the number of strains among the 8 analyzed strains that harbored mutations (regardless of the exact mutated position) within each gene. “nXm; pYq” shows the mutated position in the coding region, meaning “the ‘X’th nucleotide ‘n’ of the parent strain was changed to ‘m’ in the mutant, changing the ‘Y’th amino acid from ‘P’ to ‘Q’”. Asterisk (*) or delta (∆) indicates occurrence of a stop codon or elimination of a start codon, respectively. “X-Y del” means “the coding region from ‘X’th to ‘Y’th nucleotide in the parent strain was deleted in the mutant”, and “del-fs” indicates the occurrence of a frame shift. Note that strains are shown in order of VCM MIC, and each column is shaded white (VCM MIC = 8 μg/ml), light gray (VCM MIC = 16 μg/ml), or dark gray (VCM MIC = 32 μg/ml). Among the listed genes, the most frequently mutated SA0615 (*graS*) gene (mutated in 5 of the 8 strains) is shown in red. SA0173 (*ausA*), SA0519 (*sdrC*), and SA1584 were mutated in 3 of 4 highly resistant strains (MIC = 32 μg/ml) and are shown in blue.
