# Supplementary material for: Phenotypic and genomic comparisons of highly vancomycin-resistant Staphylococcus aureus strains developed from multiple clinical MRSA strains by in vitro mutagenesis
Source: Sci Rep. 2015 Nov 25;5:17092. doi: 10.1038/srep17092 (PMC4658547; doi:10.1038/srep17092)
Supplement: Supplementary Information [file srep17092-s1.doc]

**Phenotypic and genomic comparisons of highly vancomycin-resistant *Staphylococcus aureus* strains developed from multiple clinical MRSA strains by *in vitro* mutagenesis**

Kenichi Ishii, Fumiaki Tabuchi, Miki Matsuo, Keita Tatsuno, Tomoaki Sato, Mitsuhiro Okazaki, Hiroshi Hamamoto, Yasuhiko Matsumoto, Chikara Kaito, Tetsuji Aoyagi, Keiichi Hiramatsu, Mitsuo Kaku, Kyoji Moriya, Kazuhisa Sekimizu.

**Supplemental Information**

| Strain name | Description | Reference |
| --- | --- | --- |
| MR1-MR8 | Methicillin-resistant, clinically isolated at The University of Tokyo Hospital | This study |
| T2, T3, T5, T7, T8 | Methicillin-resistant, clinically isolated at Tohoku University Hospital | [^1^](#_ENREF_1) |
| MS9 | Methicillin-sensitive, clinically isolated at The University of Tokyo Hospital | This study |
| MSSA1 | Methicillin-sensitive, clinically isolated at Kyushu University Hospital | [^2^](#_ENREF_2) |
| Newman | Methicillin-sensitive, clinically isolated strain | [^3^](#_ENREF_3) |
| RN4220 | Methicillin-sensitive, laboratory strain | [^4^](#_ENREF_4) |
| VR1 | VCM-resistant, originating from MR1, isolated after 24 rounds of EMS/VCM selection | This study |
| VR2 | VCM-resistant, originating from MR2, isolated after 22 rounds of EMS/VCM selection | This study |
| VR3-EMS6 | VCM-resistant, originating from MR3, isolated after 6 rounds of EMS/VCM selection | This study |
| VR3-EMS10 | VCM-resistant, originating from MR3, isolated after 10 rounds of EMS/VCM selection | This study |
| VR3(-EMS20) | VCM-resistant, originating from MR3, isolated after 20 rounds of EMS/VCM selection | This study |
| VR4 | VCM-resistant, originating from MR4, isolated after 25 rounds of EMS/VCM selection | This study |
| VS5 | VCM-resistant, originating from MR5, isolated after 25 rounds of EMS/VCM selection | This study |
| VR6 | VCM-resistant, originating from MR6, isolated after 24 rounds of EMS/VCM selection | This study |
| VR7-EMS6 | VCM-resistant, originating from MR7, isolated after 6 rounds of EMS/VCM selection | This study |
| VR7-EMS10 | VCM-resistant, originating from MR7, isolated after 10 rounds of EMS/VCM selection | This study |
| VR7(-EMS22) | VCM-resistant, originating from MR7, isolated after 22 rounds of EMS/VCM selection | This study |
| VR8 | VCM-resistant, originating from MR8, isolated after 25 rounds of EMS/VCM selection | This study |
| VR-MS9 | VCM-resistant, originating from MS9, isolated after 26 rounds of EMS/VCM selection | This study |
| VR-RN | VCM-resistant, originating from RN4220, isolated after 19 rounds of EMS/VCM selection | This study |
| Mu3 | Heterogeneous vancomycin intermediate resistant strain (hVISA), clinical isolate | [^5^](#_ENREF_5) |
| Mu50 | Vancomycin intermediate resistant strain (VISA), clinical isolate | [^5^](#_ENREF_5) |

**Supplemental Table S1. *S. aureus* strains used in this study.**

| Strain | VCM | OXA | Strain | VCM | OXA |
| --- | --- | --- | --- | --- | --- |
| MR1 | 1.5 | > 256 | VR1 | 8.0 | > 256 |
| MR2 | 2.0 | > 256 | VR2 | 12 | > 256 |
| MR3 | 2.0 | > 256 | VR3 | 24 | > 256 |
| MR4 | 1.5 | 3.0 | VR4 | 32 | > 256 |
| MR5 | 1.5 | > 256 | VR5 | 8.0 | > 256 |
| MR6 | 1.5 | 8.0 | VR6 | 16 | > 256 |
| MR7 | 1.5 | > 256 | VR7 | 16 | > 256 |
| MR8 | 1.5 | > 256 | VR8 | 8.0 | > 256 |
| MS9 | 2.0 | 0.50 | VR-MS9 | 32 | 0.23 |
| RN4220 | 1.5 | 0.094 | VR-RN | 16 | 0.19 |

**Supplemental Table S2. MIC values of VCM and OXA determined by the E-test.**

MIC values were determined after 72 h incubation at 37˚C by E-test. VCM, vancomycin; OXA, oxacillin. Unit: μg/ml.

| Strain | VCM MIC (μg/ml) | Doubling time (min) |
| --- | --- | --- |
| MR3(-EMS0) | 2 | 34 |
| VR3-EMS6 | 8 | 41 |
| VR3-EMS10 | 16 | 52 |
| VR3(-EMS20) | 32 | 60 |
| VR3-L1 | 4 | 43 |
| VR3-L2 | 4 | 32 |
| MR7(-EMS0) | 1 | 27 |
| VR7-EMS6 | 8 | 39 |
| VR7-EMS10 | 16 | 40 |
| VR7(-EMS22) | 32 | 50 |
| VR7-L1 | 8 | 41 |
| VR7-L2 | 8 | 43 |
| Mu50 | 8 | 37 |
| RN4220 | 1 | 32 |

**Supplemental Table S3. MIC values of VCM and doubling time of the parent MRSA strains and VCM-resistant mutants.**

MIC values were determined after 48 h incubation at 37˚C by the micro-dilution method. The doubling time was calculated from the plotted OD_600_ values at the exponential growth phase in drug-free medium. VR3/7-L1 and L2 were isolated from large-sized colonies that appeared after growing the VR3(-EMS20) and VR7(-EMS22) strains on drug-free nutrient agar.

**[References]**

1 Aoyagi, T. *et al.* Impact of psm-mec in the mobile genetic element on the clinical characteristics and outcome of SCCmec-II methicillin-resistant Staphylococcus aureus bacteraemia in Japan. *Clinical microbiology and infection : the official publication of the European Society of Clinical Microbiology and Infectious Diseases* **20**, 912-919, doi:10.1111/1469-0691.12575 (2014).

2 Akimitsu, N. *et al.* Increase in resistance of methicillin-resistant Staphylococcus aureus to beta-lactams caused by mutations conferring resistance to benzalkonium chloride, a disinfectant widely used in hospitals. *Antimicrobial agents and chemotherapy* **43**, 3042-3043 (1999).

3 Duthie, E. S. & Lorenz, L. L. Staphylococcal coagulase; mode of action and antigenicity. *Journal of general microbiology* **6**, 95-107 (1952).

4 Peng, H. L., Novick, R. P., Kreiswirth, B., Kornblum, J. & Schlievert, P. Cloning, characterization, and sequencing of an accessory gene regulator (agr) in Staphylococcus aureus. *Journal of bacteriology* **170**, 4365-4372 (1988).

5 Hiramatsu, K. *et al.* Dissemination in Japanese hospitals of strains of Staphylococcus aureus heterogeneously resistant to vancomycin. *Lancet* **350**, 1670-1673, doi:10.1016/S0140-6736(97)07324-8 (1997).
